# Computed Tomography Scan of the Chest Is a Reliable Screening Investigation for Structural Cardiac and Pericardial Injury in Patients With Trauma

**DOI:** 10.7759/cureus.77329

**Published:** 2025-01-12

**Authors:** Chao-Kun Chen, Li-Chin Cheng, Kuo-Tai Chen

**Affiliations:** 1 Department of Thoracic Surgery, Chi Mei Medical Center, Tainan City, TWN; 2 Division of Traumatology, Department of Surgery, Chi Mei Medical Center, Tainan City, TWN; 3 Department of Emergency, Chi Mei Medical Center, Tainan City, TWN

**Keywords:** cardiac injury, computed tomography, hemopericardium, pericardial effusion, pneumopericardium

## Abstract

Background

The objective of this study was to assess the efficacy of a computed tomography (CT) scan of the chest in detecting structural cardiac and pericardial injuries.

Methods

We retrospectively analyzed data from a data registry at a trauma center between January 2015 and June 2022. This study included individuals with severe chest trauma (defined as an Abbreviated Injury Scale score of ≥3 for the chest). The assessment of cardiac injury from chest CT scans relied on the information provided in the official radiological reports. Definitive diagnoses of structural cardiac injury were confirmed on the basis of surgical findings or the diagnosis upon discharge.

Results

The chest CT scans revealed 11 cases of pericardial abnormalities: 10 (90.9%) cases of pericardial effusion in patients with blunt trauma and one (9.1%) case of pneumopericardium in a patient with a stab wound. Among these 11 cases, surgical exploration identified four structural cardiac and pericardial injuries, and three died during hospitalization. The remaining seven cases underwent nonsurgical intervention, and none exhibited any cardiac and pericardial abnormalities. The chest CT for traumatic structural cardiac and pericardial injuries had a sensitivity of 4/4 (100.0%), a specificity of 402/411 (97.8%), a positive predictive value of 4/11 (36.4%), and a negative predictive value of 404/404 (100.0%).

Conclusion

This study highlighted a high mortality rate among patients diagnosed with structural cardiac injuries, underscoring the critical importance of accurate and timely diagnostic investigations in such cases. Our findings confirmed that chest CT is a reliable screening tool for detecting structural cardiac injuries in patients with both blunt and penetrating chest trauma. However, given the relatively low positive predictive value of chest CT for structural cardiac injuries, additional diagnostic imaging or prompt surgical intervention may be necessary in cases where pericardial abnormalities are identified on chest CT to address potential occult cardiac injuries.

## Introduction

Cardiac injury often results from chest injury and is associated with high mortality risk [1-3]. Cardiac injury varies from manageable myocardial contusions, which necessitate only medical intervention, to structural abnormalities of the heart and pericardium, which require surgical measures [2,4,5]. Diagnosing cardiac injury is challenging due to its diverse symptomatic presentations. However, accurate and timely diagnosis and treatment are crucial for reducing the risks of morbidity and mortality. In routine clinical practice, a computed tomography (CT) scan of the chest is commonly employed to assess patients with chest trauma [2,3]. Pericardial abnormalities, such as hemopericardium and pneumopericardium, are considered indicative of cardiac injury [4-7]. Nonetheless, whether pericardial abnormalities detected on chest CT correspond to actual injury to the heart remains an ongoing debate [7-9].

Original research on the relationship between chest CT and cardiac injury is limited and primarily consists of retrospective studies. Most of these studies encompass all patients with cardiac injuries, rarely focusing specifically on the role of chest CT in diagnosing such injuries [10,11]. Although some case reports and case series mention findings on chest CT related to cardiac injuries, healthcare professionals require solid evidence on the diagnostic capability of chest CT for cardiac injuries [12,13]. Establishing this evidence is critical for guiding clinical decisions in trauma care.

The objective of this study was to assess the efficacy of chest CT in detecting structural cardiac and pericardial injuries.

## Materials and methods

Study design and study population

We retrospectively analyzed data from a data registry at the urban emergency department of a trauma center that is visited by more than 100,000 patients annually. The data registry encompassed the records of 22,015 patients with trauma admitted between January 2015 and June 2022.

Inclusion and exclusion criteria

This study included individuals with severe chest trauma (defined as an Abbreviated Injury Scale score of ≥3 for the chest) and excluded those lacking an official chest CT radiological report; these criteria resulted in a cohort of 415 patients. The assessment of cardiac injury from chest CT relied on the information provided in the official radiological reports. Definitive diagnoses of structural cardiac injury were confirmed on the basis of surgical findings or the final diagnosis upon discharge. Furthermore, we scrutinized the medical records of patients who died during hospitalization to identify any potentially overlooked structural cardiac injury cases.

**Figure 1:**
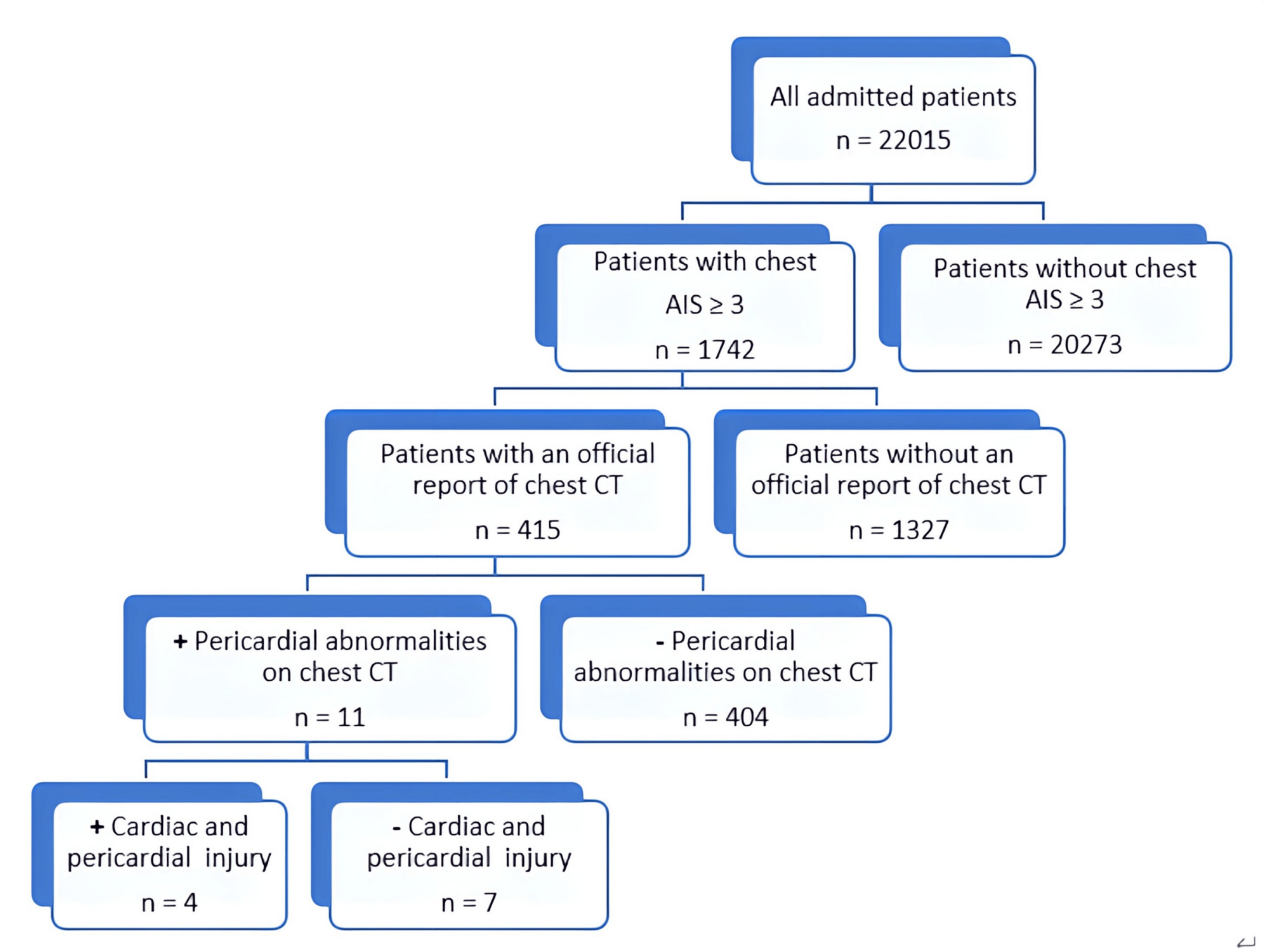
Process for selecting the study cohort and the correlation between the findings from chest CT scans and those from surgical exploration. CT, computed tomography; AIS, Abbreviated Injury Scale

Figure 1 illustrates the process for selecting the study cohort and the correlation between the findings from chest CT and those from surgical exploration.

Ethical approval

The institutional review board (IRB) of Chi Mei Medical Center approved this study (IRB number: 11304-002) and granted this study exemption from approval because the researchers used deidentified data.

Data collection and analysis

We retrospectively reviewed the data of patients with severe chest trauma. The data reviewed included demographic information (age and sex), the mechanism of trauma (blunt or penetrating), findings from the Focused Assessment with Sonography for Trauma, official chest CT reports, follow-up echocardiography during hospitalization, surgical findings, hospital course, causes of in-hospital death, and in-hospital mortality. Finally, we calculated the sensitivity, specificity, positive predictive value, and negative predictive value of chest CT for traumatic structural cardiac and pericardial injuries.

## Results

**Figure 2:**
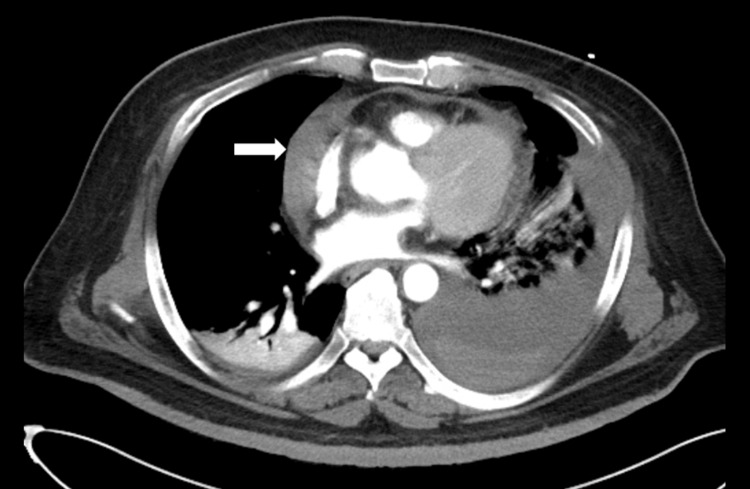
The arrow indicated fluid accumulation in pericardial space, which indicated a hemopericardium. The image was obtained from a patient in the study cohort, in whom the trauma surgeon identified a left atrial laceration during surgery.

Table 1 presents the demographic characteristics, mechanism of trauma, and mortality rates for three groups: patients with severe chest trauma, patients with pericardial abnormalities identified on chest CT, and patients with structural cardiac and pericardial injuries. Three hundred ninety-seven (95.7%) patients had sustained blunt trauma. Only 18 (4.3%) cases involved penetrative injury, namely, 16 stab wounds and two gunshot wounds. The chest CT revealed 11 cases of pericardial abnormalities: 10 (90.9%) cases of pericardial effusion in patients with blunt trauma and one (9.1%) case of pneumopericardium in a patient with a stab wound. Figure 2 presents a typical hemopericardium on images of a chest CT scan.

**Table 1:** Demographic characteristics and mortality for patients with severe chest trauma, patients with pericardial abnormalities on chest CT, and patients with structural cardiac and pericardial injury. CT: computed tomography

	Chest Abbreviated Injury Scale score of ≥3 (n = 415)	Pericardial abnormalities on chest CT (n = 11)	Structural cardiac and pericardial injury (n = 4)
Age	57.0 (44.0-67.0)	60.0 (44.0-76.0)	52.0 (40.0-64.0)
Sex (male)	271 (65.3%)	7 (63.6%)	4 (100.0%)
Penetrating injury	18 (4.3%)	1 (9.1%)	1 (25.0%)
Mortality	12 (2.9%)	4 (36.4%)	3 (75.0%)

Among these 11 cases, surgical exploration identified four structural cardiac injuries: two left atrial appendage lacerations, one left atrial laceration, and one aortic pseudoaneurysm resulting in hemopericardium. Nonsurgical interventions were pursued for the remaining seven cases. Four patients with suspected pericardial effusion underwent cardiologist-conducted transthoracic echocardiography, which excluded this condition, whereas the remaining three patients did not exhibit any cardiac and pericardial abnormalities or signs of shock throughout their hospitalization. The chest CT for traumatic structural cardiac injuries had a sensitivity of 4/4 (100.0%), a specificity of 402/411 (97.8%), a positive predictive value of 4/11 (36.4%), and a negative predictive value of 404/404 (100.0%).

Twelve patients passed away during their hospital stay. Of these patients, three died because of structural cardiac and pericardial injury, and their chest CT revealed pericardial abnormalities. The remaining nine died because of traumatic brain injury, exsanguination due to intra-abdominal injury, or infectious complications. None of these patients displayed any cardiac or pericardial abnormalities throughout their hospitalization.

Additionally, in the emergency department, 172 (41.4%) patients underwent a Focused Assessment with Sonography for Trauma investigation. None of these patients exhibited pericardial effusion, which aligned with the findings of their chest CT.

## Discussion

The results of this retrospective study indicate that chest CT can detect structural cardiac injury in patients with severe chest trauma with high sensitivity, specificity, and negative predictive value. The findings of this study corroborate the results of studies on penetrative and blunt cardiac injuries [6,14]. However, the relatively low positive predictive value suggests that pericardial effusion detected on chest CT scans may not consistently correlate with structural cardiac injury. Close observation and monitoring are practical care strategies, particularly in hemodynamically stable patients.

One patient included in this study underwent emergent surgery due to significant hemothorax and a small amount of pericardial effusion, and the surgery revealed a left atrial laceration that had been overlooked before surgery. The patient died partly because of the delayed diagnosis of cardiac injury prior to surgery and because of the time required to shift them from the lateral decubitus position for thoracoscopic surgery to the supine position for sternotomy. Consequently, we advocate considering cardiac surgery for patients with trauma exhibiting preoperative pericardial abnormalities on chest CT scans, especially when emergency surgery is required.

In addition to the early detection of structural cardiac injuries, chest CT provides critical information about concomitant injuries in patients with severe chest trauma [10,15]. These details are invaluable for trauma surgeons in formulating an appropriate surgical plan, particularly for hemodynamically unstable patients. For hemodynamically stable patients, if no significant concomitant injuries are identified on chest CT, nonoperative management or a simple pericardial window procedure may be feasible treatment options [16,17].

Esumi et al. reported a trauma patient in a state of shock caused by lacerations of the left atrial appendage and the right atrium. The only diagnostic clue was a small pericardial effusion detected on chest CT [18]. This case underscores the importance of rapid and accurate detection of pericardial effusion on chest CT in patients with cardiac injuries. The effectiveness of deep convolutional neural networks for automated chest CT analysis has already been demonstrated [19]. Implementing an automated machine learning tool could aid in identifying subtle pericardial effusions in patients with severe chest trauma, prompting further diagnostic imaging or timely surgical intervention for occult cardiac injuries.

Limitation

This study had several limitations. First, as a retrospective analysis, it may be subject to inherent biases related to data collection and study design. Second, the study was conducted in a single trauma center, which may limit the generalizability of the findings to patients in general emergency department settings. Finally, patient care for severe chest trauma in this study did not follow a standardized protocol, as therapeutic decisions were primarily made at the discretion of the trauma surgeon. This variability may introduce selection bias and impact the consistency of treatment outcomes.

## Conclusions

This study highlighted a notably high mortality rate among patients diagnosed with structural cardiac injuries, underscoring the critical importance of accurate and timely diagnostic investigations in such cases. Our findings confirmed that chest CT is a reliable screening tool for detecting structural cardiac injuries in patients with both blunt and penetrating chest trauma. However, given the relatively low positive predictive value of chest CT for structural cardiac injuries, additional diagnostic imaging or prompt surgical intervention may be necessary in cases where pericardial abnormalities are identified on chest CT to address potential occult cardiac injuries.
